# Individual, Family, and Community Characteristics Associated With COVID-19–Specific Worry and Lack of Worry Among Norwegian High School Students in First Pandemic Year

**DOI:** 10.1001/jamanetworkopen.2022.0337

**Published:** 2022-02-24

**Authors:** Jasmina Burdzovic Andreas, Geir Scott Brunborg

**Affiliations:** 1 Department of Alcohol, Tobacco, and Drugs, Norwegian Institute of Public Health, Oslo, Norway; 2 Deptment of Psychology, University of Oslo, Oslo, Norway

## Abstract

This cohort study examines the association of individual, family, and community characteristics among Norwegian high school students with COVID-19–specific worry.

## Introduction

Research on adolescent mental health during the COVID-19 pandemic tends to focus on individuals with increased anxiety levels,^1,2^ overlooking adolescents who may not be worried at all. Understanding heterogeneity of adolescents’ pandemic-specific worries may be especially relevant because these may be associated with differential compliance with health and control measures^3^ and perhaps necessitate differential public health approaches.

We investigated associations of various characteristics at the individual, family, and community levels with different types and levels of pandemic-specific worries among Norwegian high school students during the initial pandemic year. These worries included excessive worries and no worries at all.

## Methods

This cohort study was approved by the Norwegian Data Protection Authority after ethical evaluation by the National Committee for Research Ethics in the Social Sciences and the Humanities. Written parental consent was obtained for all participants. The study followed the Strengthening the Reporting of Observational Studies in Epidemiology (STROBE) reporting guideline.

We analyzed data from 2319 Norwegian high school students from the nationwide Monitoring Young Lifestyles (MyLife) study longitudinally conducted in annual assessments since autumn 2017 (ie, the time 1 baseline).^4^ Study details are provided elsewhere^4,5^ and in eMethods in the Supplement.

In late 2020 (ie, time 4), study participants reported no worry, moderate worry, or excessive worry concerning their own COVID-19 infection, infection of friends and family, and schooling. These items were adopted from the validated Pandemic Anxiety Scale questions.^1^ They are comparable with other brief measures of pandemic anxieties and worries.^3^

Risk assessments were done at time 1 (ie, 2017; baseline), time 2 (ie, 2018), time 3 (ie, 2019), and time 4 (ie, 2020). Putative factors we investigated were conceptually aligned with other factors previously found to be associated with pandemic anxiety (Table 1; eMethods in the Supplement).^1,3,6^ These included students’ prepandemic histories of mental health, physical health, academic, and other health risk (ie, presence vs absence of risk for each area); their families’ immigrant background, education level, and prepandemic history of financial risk and serious illness or death; and their communities’ urbanity, standard of living, and current COVID-19 infection rate.

**Table 1.** Participant Characteristics

Characteristic	Students, No. (%)^a^^,^^b^									
	Full sample (N = 2319)	Worries about own infection (n = 2315)			Worries about friends and family infection (n = 2317)			Worries about digital schooling (n = 2317)		
		None (n = 859 [37.1])	Moderate (n = 1261 [52.5])	Excessive (n = 195 [8.4])	None (n = 647 [27.9])	Moderate (n = 1264 [54.6])	Excessive (n = 406 [17.5])	None (n = 1000 [43.1])	Moderate (n = 945 [40.8])	Excessive (n = 372 [16.1])
**Student characteristic**										
Sex										
Male	904 (39.0)	446 (49.5)	407 (45.2)	48 (5.3)	339 (37.6)	472 (52.3)	91 (10.1)	490 (54.2)	328 (36.3)	86 (9.5)
Female	1415 (61.0)	413 (12.2)	854 (60.4)	147 (10.4)	308 (21.8)	792 (56.0)	315 (22.2)	510 (36.1)	617 (43.7)	286 (10.2)
School grade in autumn 2020										
11 (age, 16 y)	914 (39.4)	346 (37.9)	503 (55.1)	64 (7.0)	238 (26.1)	513 (56.1)	163 (17.8)	436 (47.8)	364 (39.9)	112 (12.3)
12 (age 17 y)	787 (33.9)	279 (37.9)	422 (53.8)	65 (8.3)	229 (29.1)	422 (53.6)	136 (17.3)	313 (39.8)	337 (42.8)	137 (17.4)
13 (age 18 y)	618 (26.7)	216 (35.0)	336 (54.4)	66 (10.6)	180 (29.2)	329 (53.4)	107 (17.4)	251 (40.6)	244 (39.5)	123 (19.9)
Mental health risk^c^										
No	1855 (80.0)	734 (39.7)	976 (52.7)	141 (7.6)	551 (29.7)	1022 (55.2)	280 (15.1)	839 (34.3)	787 (42.5)	227 (12.3)
Yes	464 (20.0)	125 (27.0)	285 (61.4)	54 (11.6)	96 (20.7)	242 (52.2)	126 (27.1)	161 (34.7)	158 (34.0)	145 (31.3)
Physical health risk^d^										
No	1870 (80.6)	713 (38.2)	1005 (53.8)	149 (8.0)	531 (28.4)	1028 (55.0)	310 (16.6)	823 (44.1)	756 (40.4)	289 (15.5)
Yes	449 (19.4)	146 (32.6)	256 (57.1)	46 (10.3)	116 (25.9)	236 (52.7)	96 (21.4)	177 (39.4)	189 (42.1)	83 (18.5)
Academic risk^e^										
No	1865 (80.4)	685 (36.8)	1020 (54.8)	157 (8.4)	501 (26.9)	1038 (55.7)	325 (17.4)	779 (41.8)	778 (41.8)	306 (16.4)
Yes	454 (19.6)	174 (38.4)	241 (53.2)	38 (8.4)	146 (32.2)	226 (49.9)	81 (17.9)	221 (48.7)	167 (36.8)	66 (14.5)
Other health risk^f^										
No	1505 (64.9)	557 (37.0)	825 (54.8)	123 (8.2)	407 (27.1)	834 (55.4)	263 (17.5)	663 (44.1)	619 (41.2)	222 (14.7)
Yes	281 (12.1)	104 (37.2)	151 (53.9)	25 (8.9)	85 (30.2)	139 (49.5)	57 (20.3)	103 (36.8)	120 (42.9)	57 (20.3)
Unknown	533 (23.0)	198 (37.3)	285 (53.8)	47 (8.9)	155 (29.1)	291 (54.7)	86 (16.2)	234 (43.9)	206 (38.7)	93 (17.4)
**Family characteristic**										
Immigrant status^g^										
No	1773 (76.4)	654 (36.9)	968 (54.7)	149 (8.4)	508 (28.7)	955 (53.9)	308 (17.4)	770 (43.5)	736 (41.5)	266 (15.0)
Yes	241 (10.4)	86 (36.7)	135 (56.0)	20 (8.3)	45 (18.7)	145 (60.2)	51 (21.2)	89 (37.1)	105 (43.7)	46 (19.2)
Unknown	305 (13.2)	119 (39.3)	158 (52.2)	26 (8.6)	94 (30.8)	164 (53.8)	47 (15.4)	141 (43.2)	104 (34.1)	60 (19.7)
Parental education^h^										
≥1 parent with college	1762 (76.0)	645 (36.7)	971 (55.2)	143 (8.1)	466 (26.5)	995 (56.5)	300 (17.0)	756 (42.9)	730 (42.5)	274 (15.6)
No parent with college	557 (24.0)	214 (28.5)	290 (52.2)	52 (9.3)	181 (32.5)	269 (48.4)	106 (19.1)	244 (43.8)	215 (38.6)	98 (17.6)
Financial risk^i^										
No	1993 (85.9)	754 (37.9)	1081 (54.3)	155 (7.8)	559 (28.1)	1103 (55.4)	329 (16.5)	879 (44.1)	817 (41.0)	295 (14.8)
Yes	326 (14.1)	105 (32.3)	180 (55.4)	40 (12.3)	88 (27.0)	161 (49.4)	77 (23.6)	121 (37.1)	128 (39.3)	77 (23.6)
Illness or death^j^										
No	1366 (58.9)	538 (39.4)	726 (53.1)	102 (7.5)	409 (30.0)	744 (54.5)	211 (14.5)	612 (44.8)	565 (41.4)	188 (13.8)
Yes	953 (41.1)	321 (33.8)	537 (56.5)	93 (9.8)	238 (25.0)	520 (54.6)	195 (20.5)	388 (40.8)	380 (39.9)	184 (19.3)
**Community characteristic**										
Urbanity^k^										
Rural	813 (35.1)	323 (39.8)	415 (51.2)	73 (9.0)	252 (31.0)	423 (52.0)	138 (17.0)	363 (44.7)	312 (38.4)	137 (16.9)
Urban	1506 (64.9)	536 (35.6)	846 (56.3)	122 (8.1)	395 (26.3)	841 (55.9)	268 (17.8)	637 (42.3)	633 (42.1)	235 (15.6)
Standard of living^l^										
Low	587 (25.3)	228 (38.9)	303 (51.7)	55 (9.4)	167 (28.5)	312 (53.2)	108 (18.4)	264 (45.0)	231 (39.3)	92 (15.7)
Medium	980 (42.3)	367 (37.5)	530 (54.2)	81 (8.3)	281 (28.7)	528 (54.0)	169 (17.3)	411 (41.9)	397 (40.5)	172 (17.6)
High	752 (32.4)	264 (35.1)	428 (57.0)	59 (7.9)	199 (26.5)	424 (56.4)	129 (17.1)	325 (43.3)	317 (42.3)	108 (14.4)
Infection rate (Nov 1, 2020)^m^										
Low	1044 (45.0)	423 (40.6)	532 (51.1)	87 (8.35)	327 (31.3)	540 (51.8)	176 (16.9)	486 (46.6)	413 (39.6)	145 (13.9)
High	1275 (55.0)	463 (34.3)	729 (57.3)	108 (8.5)	320 (25.1)	724 (56.8)	230 (18.1)	514 (40.4)	532 (41.8)	227 (17.8)

^a^ Percentages are out of the sample population for each variable in the first data column.

^b^ Three items relevant to high school students were selected from the Pandemic Anxiety Scale,^1^ reflecting students’ worries about infection (2 items: “How worried are you about being infected with the coronavirus?” and “How worried are you about close friends or family being infected with the coronavirus?”) and schooling (1 item: “How worried are you about digital schooling situation?”). Response options indicated none (“not worried at all”), moderate (“worried a little”), and excessive (“very worried”) COVID-19–specific worries.

^c^ Mental health risk was coded as present if the individual scored within clinical range on the Patient Health Questionnaire Adolescent version (ie, scale sum scores ≥15)^6^ at any time 1 (ie, 2017) through time 3 (ie, 2019) annual assessment.

^d^ Physical health risk was coded present if poor or very poor physical health was reported at any time 1 through time 3 annual assessment or if an asthma diagnosis was reported as part of a life history assessment at time 1.

^e^ Academic risk was coded as present if a grade point average of 3.5 or lower was reported (on a possible scale of 1 = failing to 6 = outstanding) at any time 1 through time 3 annual assessment.

^f^ Other health risk was coded as present if a learning disability (eg, attention-deficit/hyperactivity disorder or dyslexia) or other physical impairment (eg, impaired vision, hearing, or motor function) was reported as part of life history assessment at time 1.

^g^ Family immigrant background status was coded as yes, no, or unknown based on time 1 reports about the primary language spoken at home.

^h^ Parental education was coded as neither parent graduated college vs at least 1 parent graduated college based on time 4 (ie, 2020) assessment.

^i^ Family financial risk was coded as present if reported at any time 1 through time 3 annual assessment.

^j^ Serious illness or death in the family was coded present if reported at any time 1 through time 3 annual assessment.

^k^ Based on original Monitoring Young Lifestyles study sampling.

^l^ Schools included in the Monitoring Young Lifestyles study were by design drawn from municipalities with low, middle, and high standards of living within corresponding counties using Statistics Norway’s Standard of Living Index, a standardized indicator reflecting community-level characteristics (eg, rates of social security, disability payments, mortality, and unemployment).

^m^ COVID-19 cumulative incidence rates up to November 1, 2020, for each municipality were retrieved from the Norwegian Surveillance System for Communicable Diseases’ publicly available data, adjusted for municipal population size, and assigned to participants based on postal address at the time 4 and dichotomized into low (ie, ≤2 per 1000 residents, capturing the MSIS color-codes indicating the 2 lowest yellow levels) and high (>2 per 1000 residents). These infection rates in practice translated into varied suppression and containment measures at the local level.

Associations between studied risk factors and 3 types of pandemic-specific worries were examined using multinomial regressions. The reference group for each question was set as individuals with moderate worries.

## Results

A total of 2319 student participants (904 [39.0%] male students and 1415 [61.0%] female students; mean [SD] age, 17.02 [0.84] years) reported pandemic-specific worries in late 2020. Moderate worries were the most common (range, 40.8%-54.6%), but excessive worries (range, 8.4%-17.5%) and no worries (range, 27.9%-43.1%) were also reported; Table 1.

### No Worries vs Moderate Worries

Estimates from the fully adjusted multinomial models are shown in Table 2. Female sex, presence of mental health risk, and residence in a community with high infection rates were associated with decreased odds of no worry about own infection. Female sex, family immigrant background, and residence in high-infection communities were associated with decreased odds of no worry about friends and family infection, while below-college parental education was associated with increased odds. Female sex and older age (grade 12 vs grade 11) were associated with decreased odds of no worry about schooling, while the presence of academic risk was associated with increased odds.

**Table 2.** Pandemic-Specific Worries by Risk Characteristic in Regression Models

Characteristic^b^	aRRR (95% CI)^a^					
	Worries about own infection		Worries about friends and family infection		Worries about digital schooling	
	None	Excessive	None	Excessive	None	Excessive
Female sex	0.47 (0.41-0.54)	1.39 (0.98-1.97)	0.56 (0.49-0.64)	1.87 (1.41-2.49)	0.56 (0.47-0.66)	1.44 (1.15-1.80)
School grade in autumn 2020						
12 (age 17-y)	1.06 (0.83-1.37)	1.17 (0.85-1.62)	1.20 (0.96-1.49)	0.97 (0.72-1.30)	0.77 (0.63-0.94)	1.21 (0.89-1.65)
13 (age 18-y)	.96 (0.68-1.35)	1.50 (1.09-2.05)	1.21 (0.94-1.55)	0.96 (0.76-1.22)	0.85 (0.71-1.00)	1.54 (1.05-2.27)
Mental health risk	0.73 (0.58-0.91)	1.11 (0.72-1.69)	0.83 (0.59-1.14)	1.51 (1.12-2.04)	1.14 (0.96-1.35)	2.91 (2.06-4.11)
Physical health risk	0.88 (0.63-1.24)	1.03 (0.69-1.53)	0.93 (0.71-1.21)	1.11 (0.82-1.52)	0.92 (0.73-1.17)	0.81 (0.57-1.15)
Academic risk	1.02 (0.80-1.29)	0.94 (0.67-1.40)	1.21 (0.97-1.52)	1.05 (0.72-1.53)	1.29 (1.07-1.58)	0.80 (0.56-1.16)
Other health risk						
Yes	1.05 (0.75-1.46)	1.06 (0.77-1.46)	1.20 (0.87-1.64)	1.20 (0.88-1.66)	0.76 (0.54-1.06)	1.29 (0.90-1.83)
Unknown	0.98 (0.69-1.39)	1.09 (0.64-1.85)	1.02 (0.65-1.59)	0.88 (0.56-1.41)	0.87 (0.62-1.21)	0.93 (0.61-1.43)
Family immigrant status						
Yes	0.97 (0.66-1.42)	0.91 (0.54-1.55)	0.55 (0.40-0.74)	1.03 (0.68-1.56)	0.78 (0.60-1.02)	1.12 (0.85-1.47)
Unknown	1.01 (0.68-1.52)	0.99 (0.50-1.97)	0.95 (0.53-1.70)	1.08 (0.65-1.77)	1.34 (0.83-2.17)	1.84 (1.10-3.05)
No parent with college degree	1.03 (0.82-1.30)	1.14 (0.83-1.56)	1.31 (1.06-1.64)	1.24 (0.95-1.60)	1.00 (0.80-1.25)	1.14 (0.85-1.53)
Family financial risk	0.97 (0.70-1.33)	1.39 (0.90-2.15)	1.17 (0.83-1.64)	1.28 (0.89-1.85)	0.91 (0.66-1.23)	1.27 (0.85 − 1.90)
Family illness or death	0.87 (0.72-1.05)	1.14 (0.86-1.52)	0.86 (0.70-1.04)	1.19 (0.94-1.50)	.99 (0.83-1.17)	1.29 (1.05-1.59**)**
Urban community	0.87 (0.69-1.09)	.87 (0.55-1.37)	0.92 (0.77-1.12)	1.05 (0.79-1.39)	0.93 (0.75-1.17)	0.80 (0.58-1.09)
High community infection rate	0.78 (0.63-0.97)	0.98 (0.65-1.50)	0.76 (0.63-0.92)	1.002 (0.79-1.27)	0.85 (0.69-1.04)	1.40 (1.04-1.86)

Abbreviation: aRRR, adjusted relative risk ratio.

^a^ Reported are unstandardized estimates from multinomial regression models. All models accounted for school-level nesting of the original sampling strategy. The reference category in all models was moderate worries.

^b^ Reference categories were no or none for all variables, except for high school grade in autumn 2020 (reference category, grade 11), urban community (reference category, rural), and community infection rate (reference category, low). Community standard of living was not included in the fully adjusted models because there were no significant crude associations with examined outcomes in univariable models.

### Excessive vs Moderate Worries

Oldest age was associated with increased odds of excessive worries about own infection. Female sex and presence of mental health risk were associated with increased odds of excessive worries about infection of friends or family. Female sex, oldest age (grade 13 vs grade 11), presence of mental health risk, unknown family immigrant status, history of illness or death in the family, and residence in communities with high infection rates were associated with increased odds of excessive worry about schooling.

## Discussion

In this prospective cohort study, we found that most high school students from our Norwegian sample were moderately worried about COVID-19 infection and schooling during the initial pandemic year. However, many respondents also reported having no worries, and some individuals reported having excessive worries. These responses were largely associated with the current community contagion, while different participant and family prepandemic characteristics were associated with increased or decreased odds of worries or had no associations.

The study has several limitations. The results should be understood in the context of a strong governmental response and relatively low COVID-19 incidence in Norway during 2020, which may limit generalizability. Additionally, the study characteristics included relatively short-term pandemic exposure, reliance on self-reports, and a possibly nonrepresentative sample.^5^ Nevertheless, these findings may be of relevance in identification of diverse at-risk profiles (ie, those at either end of the pandemic-anxiety spectrum) during the COVID-19 pandemic and future public health crises.

## Notes

Supplement.
eMethods.
