# Supplementary material for: Individual, Family, and Community Characteristics Associated With COVID-19–Specific Worry and Lack of Worry Among Norwegian High School Students in First Pandemic Year
Source: JAMA Netw Open. 2022 Feb 24;5(2):e220337. doi: 10.1001/jamanetworkopen.2022.0337 (PMC8874346; doi:10.1001/jamanetworkopen.2022.0337)
Supplement: Supplement. — eMethods. [file jamanetwopen-e220337-s001.pdf]

Supplemental Online Content

Burdzovic Andreas J, Brunborg GS. Individual, family, and community characteristics associated with COVID-19–specific worry and lack of worry among Norwegian high school students in first pandemic year. *JAMA Netw Open*. 2022;5(2):e220337. doi:10.1001/jamanetworkopen.2022.0337

eMethods.

This supplemental material has been provided by the authors to give readers additional information about their work.

© 2022 Burdzovic Andreas J et al. *JAMA Network Open.*

eMethods.

Study design and procedures: MyLife recruited a core multi-cohort sample of 3,512 8th, 9th, and 10th graders from 33 middle schools throughout Norway in Oct-Dec 2017, and has so far completed annual follow-ups in Oct-Dec of 2018 (T2), 2019 (T3), and 2020 (T4). At each assessment, the entire core sample was invited to complete e-questionnaires either in school (while in middle school) or in their own time (once in high school). The number of respondents was 2,975 (84.7% of the core sample) at T1 2017 baseline, 2,875 (81.8%) at T2 2018 follow-up, 2,651 (75.5%) at T3 2019 follow-up, and 2,323 (66.1%) at T4 2020 follow-up. Only 86 students did not participate in any of the four assessments so far; i.e., by 2020 , we had at least one data point for all but 86 of the 3,512 students from the core sample. More importantly, we had at least one T1-T3 data point for all 2,319 students who completed T4 2020 assessment and reported pandemic-related worries.

Sample: After excluding the cases without any responses on the key pandemic-worries outcomes and without Norwegian residence address at T4 (*n* = 4), our analytical sample included 2,319 high school students who completed T4 assessment in late 2020.

Measures:

Pandemic-specific worries: Three items relevant to high school students were selected from the Pandemic Anxiety Scale[1]. These reflected worries about the infection (2 items; "How worried are you about being infected with the Coronavirus?" and "How worried are you about close friends or family being infected with the Coronavirus?") and schooling (1 item; "How worried are you about the digital schooling situation?"). The response options for all three indicators were "not at all" (coded "no worries"), "a little worried" (coded "moderate worries"), and "very worried" (coded "excessive worries"). These and similar short measures of pandemic-specific anxieties and worries have been successfully used in previous research[3, 5].

Individual characteristics: In addition to their *sex* and *school grade in Autumn 2020*, we used T1-T3 (2017-19) pre-pandemic assessments to obtain recent histories of students':

© 2022 Burdzovic Andreas J et al. *JAMA Network Open.*

a) *mental health problems*, coded present if scored within clinical range on the Patient Health Questionnaire (PHQ-9; Adolescent version where scale sum scores $\geq$ 15 are indicative of major depressive disorder)[6] at any T1-T3 assessment;

b) *physical health problems*, coded present if reported "poor" or "very poor" physical health at any T1-T3 assessment, or reported asthma diagnosis as part of a life history assessment at T1 baseline in 2017;

c) *academic problems*, coded present if reported GPA of 3.5 or lower (on a possible scale of 1=failing to 6=outstanding) at any T1-T3 assessment;

d) *learning disabilities or any other chronic health impairments*, coded present if reported learning disability (i.e., ADHD, dyslexia, etc.) or other physical impairment (i.e., impaired vision, hearing, motor function, etc.) as part of the life history assessment at T1 baseline in 2017.

Family characteristics: We accounted for *immigrant background* (coded: yes/no/unknown) based on T1 baseline reports about the language spoken at home and for *parental education* based on T4 reports (coded: neither parent graduated college vs. at least one or both parents graduated college or greater). In addition, we used T1-T3 (2017-19) pre-pandemic reports to obtain histories of *financial problems in the family* (*coded* present if reported at any T1-T3 assessment) and *serious illness and/or death in the family* (coded present if reported at any T1-T3 assessment).

Community characteristics: We accounted for the municipal *urbanity* (coded: rural vs. urban) and *standard of living* using Statistics Norway's Standard of Living Index (SLI) -- a standardized indicator reflecting community-level characteristics (e.g. rates of social security, disability payments, mortality, and unemployment). The MyLife study sampled schools from both urban and rural municipalities, and from low, middle, and high standard of living municipalities within corresponding counties[4].

Finally, we accounted for the *current infection rates* in students' municipalities of residence. The COVID-19 cumulative cases up to November 1, 2020 for each municipality were retrieved from the Norwegian Surveillance System for Communicable Diseases' (MSIS) publicly available data (https://www.covid19data.no/) and assigned to participants based on their postal address at the T4

© 2022 Burdzovic Andreas J et al. *JAMA Network Open.*

2020 assessment. For ease of analyses, these cumulative incidence rates were dichotomized into low (≤ 2 per 1,000 residents; capturing the MSIS color-codes indicating the two lowest "yellow" levels) and elevated (>2 per 1,000 residents; capturing the MSIS color-codes indicating the top three "orange-red" levels). These infection rates in practice translated into varied suppression and control measures at the local level[5].

All putative predictors were conceptually aligned with other factors previously associated with pandemic anxiety[1, 3] and were additionally expanded to take into consideration the specific worries (i.e., history of academic problems in relation to schooling-specific worries for example) and rarely considered real-life conditions (i.e., community contagion).

Statistical Analyses: Missing values on covariates were conservatively classified into the no-risk category when the proportion of missing cases was under 5%. This strategy was applied to participants with missing information on mental health (44 case; 1.9%), physical health (31 cases, 1.3%), academic achievement (73 cases, 3.1%), and severe illness or death in the family (31 cases, 1.3%). Missing values, if any, on the remaining covariates were classified into a dummy (unknown) category and included as such in all analyses to prevent the loss of data. Missing values on outcomes (4 non-responses on the item asking about worrying about own infection, and the 2 non-responses on the remaining items) were not replaced, resulting in a slightly varied analytical *n*'s.

Associations between the three examined indicators of pandemic-specific worries and all predictors were examined first with the crude univariable, and then with fully adjusted multinomial regression models where all predictors were included simultaneously. All estimates from the multinomial regressions were reported as unstandardized Relative Risk Ratios (RRR, commonly interpreted as odds ratios) with 95% CI. Because we were primarily interested in factors associated with worrying either excessively or not at all about the pandemic, the response category of "moderate worries" was the reference category in all analyses.

All analyses were conducted in Stata statistical software version 15.0; robust standard errors were estimated with the vce (cluster) option, which accounted for school-level nesting associated

© 2022 Burdzovic Andreas J et al. *JAMA Network Open.*

with the original school-based sampling. P values were 2-sided, and statistical significance was set at $P < .05$; all estimates were reported with corresponding 95% CI. Data were analyzed from March to December 2021.

© 2022 Burdzovic Andreas J et al. *JAMA Network Open.*
